# A novel *PSMB8* isoform associated with multiple sclerosis lesions induces P-body formation

**DOI:** 10.3389/fncel.2024.1379261

**Published:** 2024-05-15

**Authors:** Benjamin C. Shaw, Jessica L. Williams

**Affiliations:** ^1^Department of Neurosciences, Lerner Research Institute Cleveland Clinic, Cleveland, OH, United States; ^2^Brain Health Research Institute, Kent State University, Kent, OH, United States

**Keywords:** nonsense-mediated decay, neuroinflammation, alternative splicing, multiple sclerosis, astrocytes

## Abstract

**Introduction:**

Multiple sclerosis (MS) is an inflammatory and demyelinating disease of the central nervous system (CNS). Current therapies primarily target the inflammatory component of the disease and are highly effective in early stages of MS while limited therapies have an effect in the more chronic progressive stages of MS where resident glia have a larger role. MS lesions tend to be inflammatory even after the initial peripheral immune cell invasion has subsided and this inflammation is known to cause alternative splicing events.

**Methods:**

We used qPCR of normal-appearing white matter and white matter lesions from postmortem MS tissue, *in vitro* studies, and immunostaining in MS tissue to investigate the alternative splicing of one gene known to be important during recovery in an animal model of MS, *PSMB8*.

**Results:**

We found a novel, intron-retained isoform which has not been annotated, upregulated specifically in MS patient white matter lesions. We found that this novel isoform activates the nonsense-mediated decay pathway in primary human astrocytes, the most populous glial cell in the CNS, and is then degraded. Overexpression of this isoform in astrocytes leads to an increased number of processing bodies *in vitro*, the primary site of mRNA decay. Finally, we demonstrated that MS white matter lesions have a higher burden of processing bodies compared to normal-appearing white matter, predominantly in GFAP-positive astrocytes.

**Discussion:**

The increase in alternative splicing of the *PSMB8* gene, the stress that this alternative splicing causes, and the observation that processing bodies are increased in white matter lesions suggests that the lesion microenvironment may lead to increased alternative splicing of many genes. This alternative splicing may blunt the protective or reparative responses of resident glia in and around white matter lesions in MS patients.

## Introduction

Multiple sclerosis (MS) is a chronic inflammatory, neurodegenerative disease of the central nervous system (CNS) (Compston and Coles, 2008). Histologically, MS lesions are characterized by peripheral immune cell infiltration, demyelination, and glial cell activation (Trapp and Nave, 2008). The most common clinical subtype of MS is relapsing remitting MS (RRMS). Patients with RRMS exhibit periods of exacerbated symptoms such as paresthesia, vision deficits, and muscle weakness, followed by periods of partial to complete recovery (Compston and Coles, 2008). Most of these patients will develop secondary progressive MS (SPMS), and patients in this stage of MS exhibit markedly less recovery between relapses (Hurwitz, 2009). A small proportion of MS patients will initially present with a progressive phenotype, termed primary progressive MS (PPMS), characterized by constant disability accumulation with no clear remission (Thompson et al., 1991; Confavreux et al., 2000). At any stage of the disease, patients can experience disability progression independent of relapse activity, which is the most common form of disability accumulation across all traditional MS subtypes (Müller et al., 2023). Patients with RRMS, typically characterized by a significant inflammatory component, respond well to current disease-modifying therapies, such as ocrelizumab, fingolimod, and glatiramer acetate, which are immunomodulatory (Wingerchuk and Carter, 2014). In progressive MS, however, these immune cell-targeting drugs have limited efficacy indicating that disability is perpetuated by CNS resident cells (Miller and Leary, 2007; Feinstein et al., 2015).

Astrocytes are the most abundant glial cell in the CNS and are responsible for the formation of the glial scar in MS lesions (Brosnan and Raine, 2013). Traditionally thought of as quiescent support cells, recent studies have highlighted their importance in both protection from and induction of neuroinflammation (Lee et al., 2023). Astrocytes are exquisitely responsive to immune signaling molecules, including interferons, commonly found in MS lesions (Daniels et al., 2017; Williams et al., 2020). Specifically, interferon-γ (IFNγ) upregulates the immunoproteasome within astrocytes as a protective mechanism in a murine model of MS, experimental autoimmune encephalomyelitis (EAE) (Smith et al., 2020). The immunoproteasome consists of multiple subunits–LMP7 (*PSMB8*), LMP2 (*PSMB9*), and MECL-1 (*PSMB10*)–which are induced by interferons and assemble sequentially (Griffin et al., 1998; Heink et al., 2005; Orre et al., 2013). Once assembled, the immunoproteasome is responsible for the proteolysis of polyubiquitinated and oxidatively damaged proteins (Ebstein et al., 2012).

The immunoproteasome is upregulated in MS lesions, especially in astrocytes, and inhibition of the immunoproteasome exacerbates EAE (Smith et al., 2020). Counterintuitively, activity of the immunoproteasome is decreased in MS lesions despite its protein-level upregulation (Zheng and Bizzozero, 2011). Alternative splicing can lead to a loss of function for genes critical in regulating other diseases, such as *TREM2* in Alzheimer’s disease (Kiianitsa et al., 2021; Shaw et al., 2022). Given the sequential assembly of the immunoproteasome (Griffin et al., 1998), wherein LMP7 is the first subunit incorporated followed by LMP2 and then MECL-1, disruption of either LMP7 protein or its transcript, *PSMB8*, may lead to an incomplete or inefficient induction. Poor induction of the immunoproteasome may therefore lead to further decreases in immunoproteasome activity and thus increased lesion activity. Further, inflammation induces alternative splicing of many mRNAs and alternative splicing frequently leads to premature stop codon insertion which activates the nonsense-mediated decay (NMD) pathway and induces processing bodies (P-bodies) (Janssen et al., 2020; Robinson et al., 2021). Thus, the inflammation which occurs in an MS lesion may lead to alternative splicing of critical damage control mechanisms limiting the potential recovery after CNS insult. To date, however, no studies have examined alternative splicing in CNS tissue at patient lesion sites during MS.

To address this, we focused our attention on the immunoproteasome gene *PSMB8*. This gene has a pair of mutually exclusive exons, exon 1A and exon 1B. Importantly, only the protein which results from translation of exon 1B-containing *PSMB8* can efficiently incorporate into the mature immunoproteasome (Griffin et al., 1998). While previous reports have found that inflammation can drive alternative first exon usage (Robinson et al., 2021), we found no difference in the proportion of exon 1A versus exon 1B usage in MS white matter lesions (WML) compared to normal-appearing white matter (NAWM). We did, however, uncover a previously unreported and unannotated transcript of *PSMB8* which retains intron 2 and is upregulated in WMLs. Using primary human cortical astrocytes, we found that this intron 2 retained *PSMB8* transcript (*i2R-PSMB8*) activates the NMD pathway and is then degraded. Further, overexpression of *i2R-PSMB8* increases the number of intracellular P-bodies *in vitro*. Finally, we observed a substantial increase in overall P-body burden within astrocytes among other cells within MS lesions, potentially indicating a dysregulation in splicing of many genes within the MS WML.

## Materials and methods

### Human tissue, RNA isolation, and cDNA conversion

MS brain tissues were obtained from The Cleveland Clinic according to the established rapid autopsy protocol approved by the Cleveland Clinic Institutional Review Board (Chomyk et al., 2017). Patients or their relatives provided informed consent in the form of an advanced directive as part of an institutional review board-approved protocol. Tissues originating from MS cases used for qRT-PCR and immunohistochemistry are described in Table 1. Tissues were obtained, lesion types characterized, and RNA from human cerebral tissue was isolated and converted to cDNA as previously described (Dutta et al., 2006). Paired WML and NAWM were used for qRT-PCR analysis.

**Table 1:** Patient demographics.

Patient ID	Sample Type	Age (yrs)	Sex	MS Type	EDSS	Postmortem Interval (hrs)	Disease Duration (Yr)
31	NAWM, WML	60	F	Secondary Progressive MS	9	7.83	29.5
82	NAWM, WML	51	F	Primary Progressive MS	7.5	6.92	14.9
88	NAWM, WML	57	M	Secondary Progressive MS	6.5	9.67	27.5
144	NAWM, WML	38	M	Secondary Progressive MS	7	12	17.9
167	NAWM, WML	59	F	Secondary Progressive MS	7.5	9.5	NA
170	NAWM, WML	48	F	Relapsing Remitting MS	7.5	10	NA

### End-point and qRT-PCR assays

Within patient WML and NAWM cDNA samples were amplified using a forward primer against exon 1B (5’-GCTCGGACCCAGGACACTAC-3′) and a reverse primer against exon 6 (5’-GAGAACACGCAGAAGATGCAC-3′) with AccuStart II PCR Supermix (QuantaBio) for an end-point PCR assay. Samples were amplified using an initial 1 min denaturation at 95°C, followed by cycles of 15 s at 95°C, 15 s at 57.3°C, and 2 min at 72°C for 40 cycles, followed by a 10 min final extension at 72°C before resolving on a 5% polyacrylamide tris-borate-EDTA (TBE) gel (Bio-Rad) at 35 V. Gel bands were excised, reamplified, and purified using a post-PCR cleanup kit (New England Biolabs) per manufacturer’s instructions. Purified DNA was sequenced commercially (Azenta) to identify excised bands. For qRT-PCR assays, cDNA was amplified using a forward primer in exon 1B (5’-GCTCGGACCCAGGACACTAC-3′) and a reverse primer in exon 2 (5’-GTTCCTTTCTCCGTCCCCAC-3′) to quantify total exon 1B-containing *PSMB8*. Intron 2 retention was quantified with a forward primer in intron 2 (5’-CCCTTCTCTCCCAAAGCTCC-3′) and reverse primer in exon 3 (5’-GTGCCAAGCAGGTAAGGGTT-3′). *GAPDH* was used as a housekeeping gene and quantified (5’-GAAGGTGAAGGTCGGAGTC-3′ and 5’-GAAGATGGTGATGGGATTTC-3′). All qRT-PCR assays used PowerSYBR master mix (Applied Biosystems), and assays were performed according to manufacturer’s instructions in duplicate and quantified using the double-delta Ct method. Quantification of astrocytic reactivity markers (*GBP2*, *GGTA1*, *B3GNT5*, *CD14*, *PTX3*, *S100A10*, *TGM1*, *CD44*, *GFAP*, *S1PR3*, *TIMP1*, *CCL2*, *CCL5*, *CXCL12*) was performed as described above using the primers described in Supplementary Table S1.

### Cell culture and transfections

Primary human cortical astrocytes (ScienCell) were obtained commercially and expanded in astrocyte media (ScienCell). Cells were cultured at 37°C in 5% CO_2_ and split at approximately 80% confluency until passage 3. Upon reaching passage 3, cells were frozen using Recovery Cell Freezing Media (Gibco). Cells were thawed prior to experiments and transfected during passage 4. Vectors encoding either intron 2 retained *PSMB8* (*i2R-PSMB8*) or the canonical full-length transcript (*FL-PSMB8*) in pcDNA3.1 were obtained from GenScript, outgrown in DH5α *E. coli* (New England Biolabs), and midi-prepped using a Plasmid Plus Midi kit (Qiagen) per manufacturer’s instructions. Vector maps are provided as Supplementary Figures S1, S2. Vectors were transfected using a Lonza Amaxa Nucleofector 4 device, with kit P3 and protocol DR114, at a ratio of 6 μg DNA per 10^6^ cells. This resulted in a 50–55% transfection efficiency (Supplementary Figure S3).

### Protein isolation and western blotting

Cells were cultured in 6-well plates after transfection at 80% confluency and incubated for 24 h post-transfection. Cells were then washed three times with PBS and lysed with IP lysis buffer (25 mM Tris–HCl, 150 mM NaCl, 1 mM EDTA, 1% Nonidet P-40, 5% glycerol) with 1% protease-phosphatase inhibitor (Pierce). Debris was pelleted at 15,000 RCF for 15 min at 4°C and protein concentration determined using a BCA assay (Invitrogen). Protein (50 μg per sample) was reduced using β-mercaptoethanol, denatured using SDS loading buffer (Bio-Rad) and heat denatured at 95°C for 10 min before loading onto a 4–15% Tris-glycine gel (Bio-Rad) and electrophoresed at 125 V. Gels were transferred to PVDF membranes using a Turbo transfer system (Bio-Rad). Membranes were blocked with 3% bovine serum albumin in Tris-buffered saline with 0.05% Tween-20 (TBS-T) for 1 h at room temperature and blotted with an antibody against phosphorylated upstream frameshift protein 1 (phospho-UPF1) (pUPF1; 1:1000, Cell Signaling Technologies) overnight at 4°C. Membranes were then washed three times with TBS-T prior to incubating with a goat anti-rabbit horseradish peroxidase (HRP) conjugated antibody (Invitrogen). Chemiluminescence was detected using an enhanced chemiluminescence kit (Bio-Rad) and a ChemiDoc MP system (Bio-Rad). After imaging, membranes were stripped twice for 10 min each using stripping buffer (200 mM glycine, 3.5 mM SDS, 1% Tween-20, pH 2.2), blocked with 5% non-fat milk, and blotted overnight at 4°C with an antibody against total UPF1 (1:1000, Cell Signaling Technologies). Membranes were again washed three times with TBS-T prior to incubating with a goat anti-rabbit HRP conjugated antibody (Invitrogen), and chemiluminescence detected as before.

### *PSMB8* isoform stability

Stability of mRNA isoforms was assayed as previously described (Zajac et al., 2023). Briefly, primary human cortical astrocytes (ScienCell) were cultured to passage 4 and stimulated with 50 μg/mL cycloheximide (CHX; Invitrogen) or dimethylsulfoxide (DMSO) vehicle control for 0, 1, 4, or 8 h. The assay was performed in triplicate per time point and treatment. At the designated time point, cells were lysed and RNA was collected using an RNeasy isolation kit (Qiagen) per manufacturer’s instructions. Resultant RNA was converted to cDNA using 500 ng total RNA and a high-capacity reverse transcription kit (Applied Biosystems) with final concentrations of reagents as follows: 1.75 mM MgCl_2_ (Invitrogen), 2.00 mM dNTPs (Applied Biosystems), 2.50 μM random hexamers (Invitrogen), 2.50 μM oligo(dT) (Invitrogen), 1 U/μL RNAse inhibitor (Applied Biosystems), 5 mM dithioerythritol (Invitrogen), 2.50 U/μL reverse transcriptase (Applied Biosystems). The 20 μL reactions were heated to 37°C for 30 min then heat-inactivated at 95°C for 5 min before storage at -20°C until qPCR was performed.

### Immunocytochemistry and imaging of transfected cells

Transfected cells were washed once with PBS then fixed in 4% paraformaldehyde for 30 min. Cells were blocked and permeabilized in 10% normal goat serum and 0.1% Triton-X100 in PBS for 1 h. Cells were then stained with a knockout-validated antibody against enhancer of mRNA decapping 4 (EDC4) (1:200, Abcam) (Hallacli et al., 2022) at room temperature for 1 h before washing three times with PBS. Secondary antibody (Invitrogen) was then applied for 1 h, slides washed three times with PBS, and incubated with DAPI solution (Invitrogen) to stain nuclei. Slides were coverslipped with Prolong Glass mounting media (Invitrogen) and stored at -20°C until imaged using a Zeiss LSM800 confocal microscope. ImageJ was used to automate EDC4^+^ puncta counts in GFP^+^ cells to remove bias.

### Immunohistochemistry of human MS tissue

Paraformaldehyde-fixed tissue sections (30 μm thick) were obtained from the Cleveland Clinic rapid autopsy program. Antigen retrieval was performed using 10 μM citrate buffer and boiling briefly. Free-floating sections were permeabilized with 2% Triton-X100 in PBS for 30 min then blocked with 5% normal goat serum and 0.3% Triton-X100 for 1 h. Sections were then incubated in primary antibody against major histocompatibility complex class II (MHCII; 1:50, Abcam), myelin basic protein (MBP; 1:200, Abcam), glial fibrillary acidic protein (GFAP; 1:50, Invitrogen), and EDC4 (1:40, Abcam) for 3 days. Sections were then washed in PBS with 0.3% Triton-X100 three times and incubated with secondary antibodies (Invitrogen) conjugated to AlexaFluor 405, 488, 594, or 647 for 1 h at room temperature. Slides were then washed three times and treated with TrueBlack lipofuscin quencher (Biotium) per manufacturer’s instructions. Sections were mounted using Prolong Glass (Invitrogen) and stored at -20°C until imaged using a Zeiss LSM800 confocal microscope. Cell bodies filled with EDC4 were counted by a blinded observer.

### Statistical analyses

Quantitative PCR data using the double-delta Ct method were analyzed using a one-sample *t* test. Western blot, puncta count, and cell body count data were analyzed using a two-sample *t* test. Isoform stability qPCR data were analyzed by two-way ANOVA.

## Results

### Discovery of an alternative splice variant in *PSMB8*

**Figure 1:**
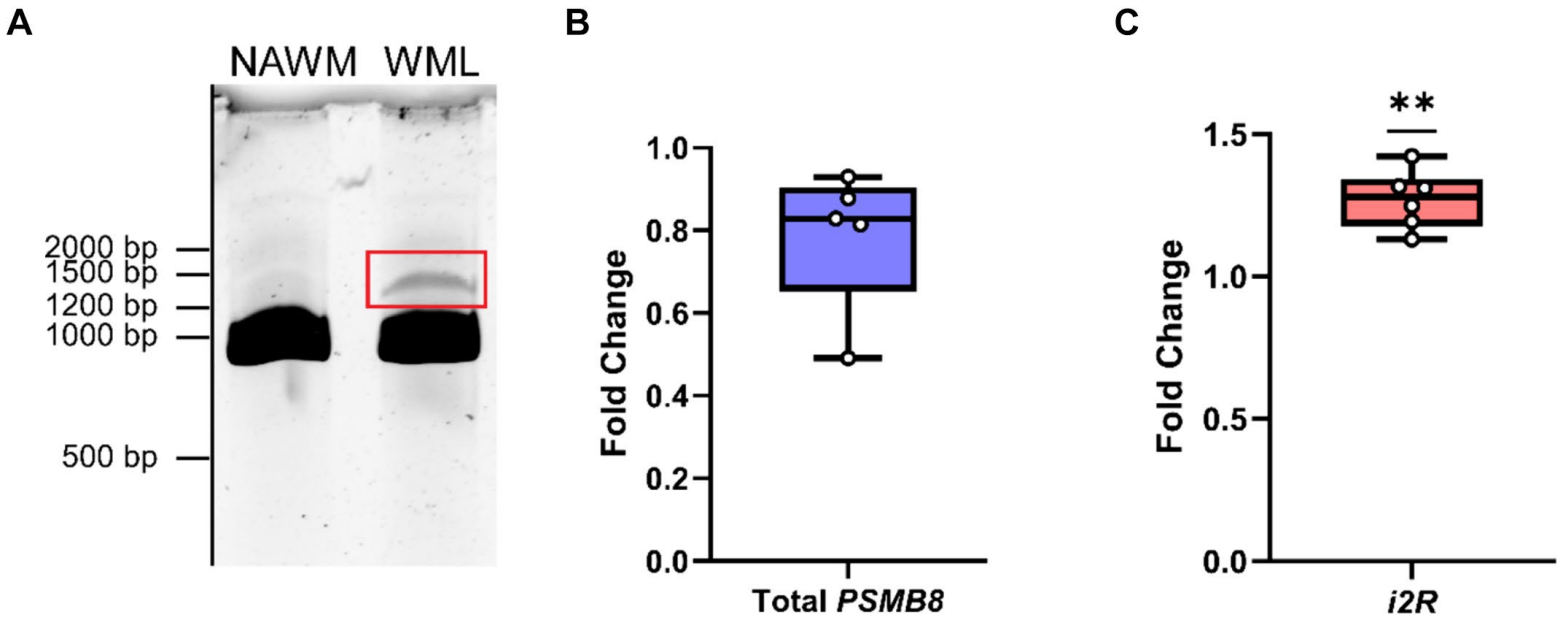
*PSMB8* undergoes alternative splicing in the MS WML. **(A)** Representative gel image of cDNA amplified with primers corresponding to exon 1B and exon 6 of *PSMB8*. cDNA was amplified from NAWM (left lane) or WML (right lane) to visualize the canonical PSMB8 isoform (918 bp) and a higher molecular weight isoform (1,076 bp, red box). **(B)** qPCR for total exon 1B was performed on cDNA from NAWM and WMLs. *PSMB8* expression is shown as a fold change calculated from the ΔΔCt of *PSMB8* to *GAPDH* and WML to NAWM. One sample was excluded due to lack of amplification in *GAPDH*. **(C)** *i2R-PSMB8* was quantified in the same samples. The *i2R-PSMB8* expression is shown as a fold change calculated from the ΔΔCt of *i2R-PSMB8* to total exon 1B containing *PSMB8* and WML to NAWM. Total exon 1B-containing *PSMB8* was used for normalization rather than *GAPDH* as it is a better representation of how the splicing changes within the lesion. Data representative of two replicate experiments from the same cDNA. *n* = 5–6 patients with matched NAWM and WML samples. One WML was analyzed per patient. Significance was determined using a one-sample *t*-test. **p < 0.01.

To address whether any novel splice variants of the functional exon 1B-containing *PSMB8* exist, we amplified cDNA from MS patients derived from either NAWM or WMLs with a forward primer targeting exon 1B and a reverse primer targeting exon 6. After amplification and polyacrylamide gel electrophoresis, the canonical isoform of *PSMB8* was highly expressed in both NAWM and WMLs. Additionally, a higher molecular weight band indicating a larger transcript was apparent in WMLs (Figure 1A). This band was excised, reamplified, and sequenced. Sequencing revealed that this novel isoform retains intron 2, leading to an additional 158 bp in the resultant mRNA, consistent with the molecular weight shift observed in WMLs (Figure 1A). This novel isoform is hereafter referred to as intron 2 retained or *i2R-PSMB8*. We did not observe a statistically significant change in total *PSMB8* expression in the WMLs compared to NAWM (Figure 1B; *p* > 0.05). By contrast, qPCR using primers targeting intron 2 and exon 3 revealed that intron 2 retention was significantly higher in WMLs compared to NAWM relative to total exon 1B expression (Figure 1C; *p* = 0.0013). While the band we identified as *i2R-PSMB8* in Figure 1A appears to represent a small amount of the total *PSMB8*, we hypothesize that this isoform is predicted to undergo NMD and is likely highly underrepresented on the gel image. These data indicate that in the lesion microenvironment, *PSMB8* splicing is dysregulated and may contribute to increased cellular stress.

### *i2R-PSMB8* enhances P-body formation in astrocytes

**Figure 2:**
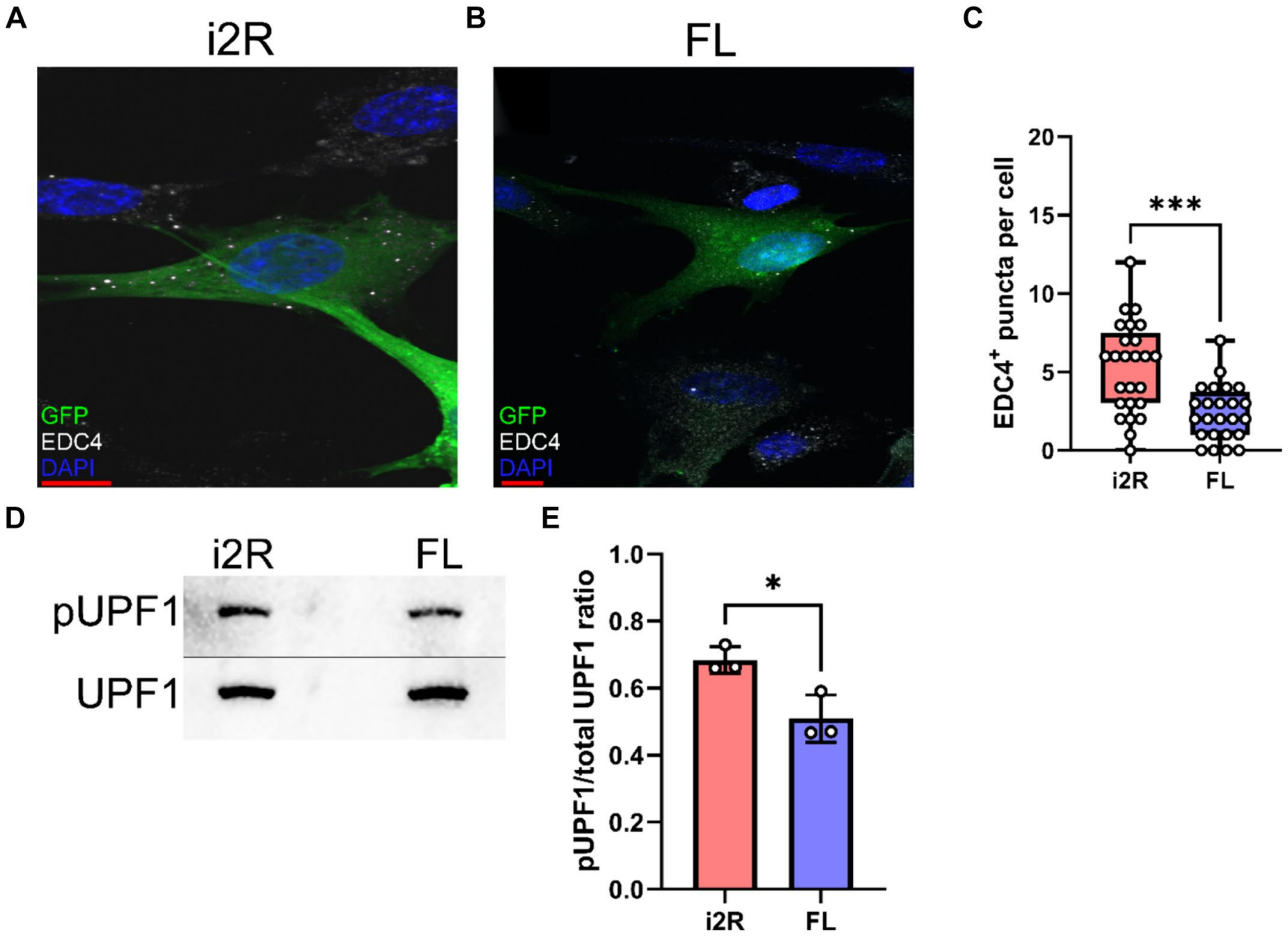
*i2R-PSMB8* induces P-body formation and leads to phosphorylation of UPF1. Human primary cortical astrocytes were transfected with vectors encoding either **(A)** intron 2 retained (i2R) or **(B)** full-length (FL) *PSMB8* and co-transfected with a vector encoding green fluorescent protein (GFP) to identify positively transfected cells. **(C)** GFP^+^ cells were identified and the automated counting of the number of EDC4^+^ puncta (white) was performed using ImageJ. Scale bars (red) are 10 μm. Data representative of two independent experiments. Each point represents an individual transfected GFP^+^ cell. **(D)** Representative western blot images of phospho-UPF1 (top) and total UPF1 (bottom) of lysates from primary human cortical astrocytes transfected with vectors encoding either intron 2 retained (i2R) or full-length (FL) *PSMB8*. **(E)** The ratio of pUPF1 to total UPF1 was quantified. Data are from three independent experiments. Significance was determined by unpaired *t* test. *p < 0.05; ***p < 0.001.

Since the *i2R-PSMB8* isoform has an additional 158 bp inserted after exon 2, there is a frameshift and resultant premature stop codon in exon 3. Given that *PSMB8* has six exons, we hypothesized that the *i2R-PSMB8* isoform would undergo NMD. P-bodies are the primary sites of mRNA decay after being targeted through the NMD pathway (Teixiera et al., 2005). Increases in NMD are correlated with increased size and number of P-bodies (Teixiera et al., 2005; Chang and Tarn, 2009; Chuang et al., 2013; Di Stefano et al., 2019), which can be quantified using EDC4, a critical component of P-body formation. To test this hypothesis, we cloned the *i2R-PSMB8* and *FL-PSMB8* transcripts and transfected these vectors into human primary cortical astrocytes in glass chamber slides and co-transfected with a vector encoding green fluorescent protein (GFP) to mark transfected cells (Figures 2A,B). At 24 h post-transfection, cells were fixed and labeled for EDC4. Notably, the number of EDC4^+^ puncta within GFP^+^ cells was significantly higher in the *i2R-PSMB8* transfected astrocytes compared to those transfected with *FL-PSMB8* (Figure 2C; *p* = 0.0003). No differences in actin morphology (data not shown) or expression of a subset of reactive astrocyte phenotypic markers, and cytokine and chemokine transcripts were observed between groups at 24 h post-transfection (Supplementary Figure S4). This indicates the *i2R-PSMB8* transcript may lead to increased NMD. To test this, we again transfected primary human cortical astrocytes with the *i2R-PSMB8* or *FL-PSMB8* vectors and quantified the phosphorylation of UPF1, an NMD initiating event (Kashima et al., 2006). At 24 h post-transfection, we assessed the ratio of pUPF1 to total UPF1 via western blot (Figure 2D) and found that there was significantly enhanced activation of UPF1 in cells transfected with *i2R-PSMB8* compared to *FL-PSMB8* (Figure 2E; *p* = 0.0196).

### The *i2R-PSMB8* transcript undergoes NMD in astrocytes

**Figure 3:**
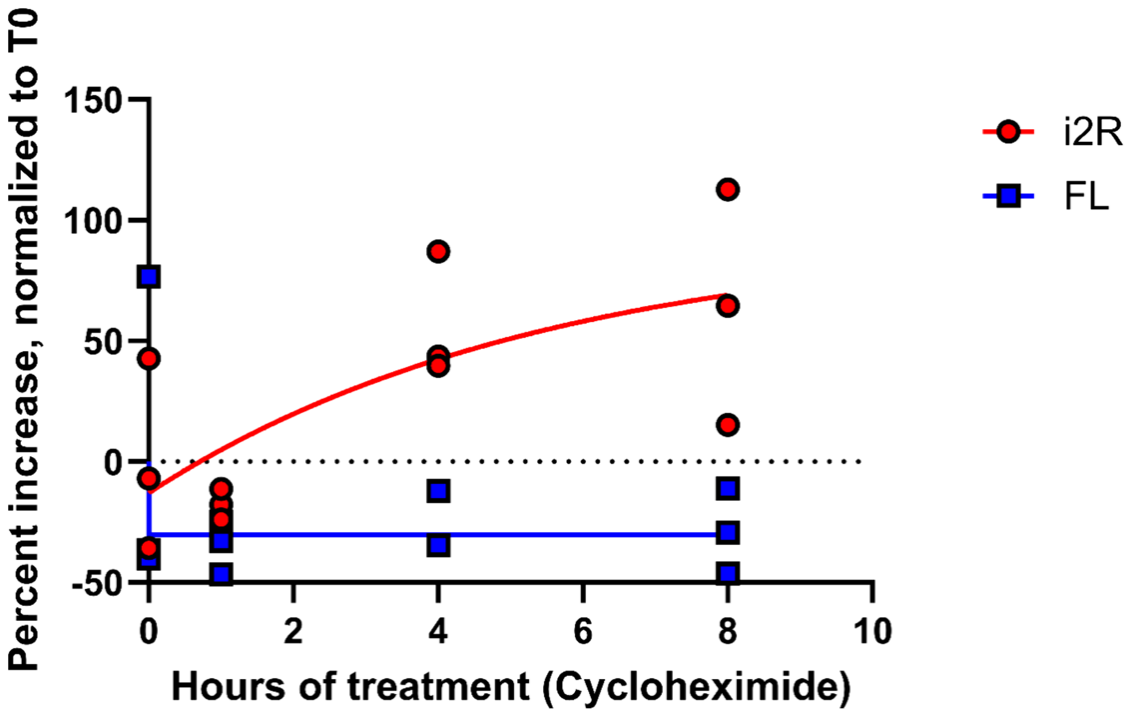
i2R-PSMB8 undergoes NMD in astrocytes. qPCR measurements of intron 2 retained (i2R) or full-length (FL) PSMB8 transcript after the addition of cycloheximide. Data are normalized to time 0 and are representative of two independent experiments. Significance was determined by two-way ANOVA. F_3,16_ = 18.09, *p* < 0.0001.

The NMD pathway is dependent on first-pass translation (Behm-Ansmant and Izaurralde, 2006), and inhibiting translation through treatment with cycloheximide causes transcripts typically targeted for NMD to become more stable (Zajac et al., 2023). To directly test whether the *i2R-PSMB8* transcript undergoes NMD, we treated primary human cortical astrocytes with either cycloheximide (50 μg/mL) or DMSO as a vehicle control and incubated for up to 8 h. Total RNA was isolated at 0, 1, 4, and 8 h post-treatment and converted to cDNA for qPCR. Subsequent qPCR revealed a time-and drug-dependent interaction (Figure 3; *F_3,16_ =* 18.09*, p* < 0.0001), indicative of an increase in *i2R-PSMB8* transcript in the cycloheximide-treated astrocytes, suggesting that the *i2R-PSMB8* transcript undergoes NMD. The *FL-PSMB8* transcript did not change over time in the presence of cycloheximide, indicating our assay is specific for nonsense-mediated decay transcripts (Figure 3).

### P-body load is increased in postmortem MS lesions

**Figure 4:**
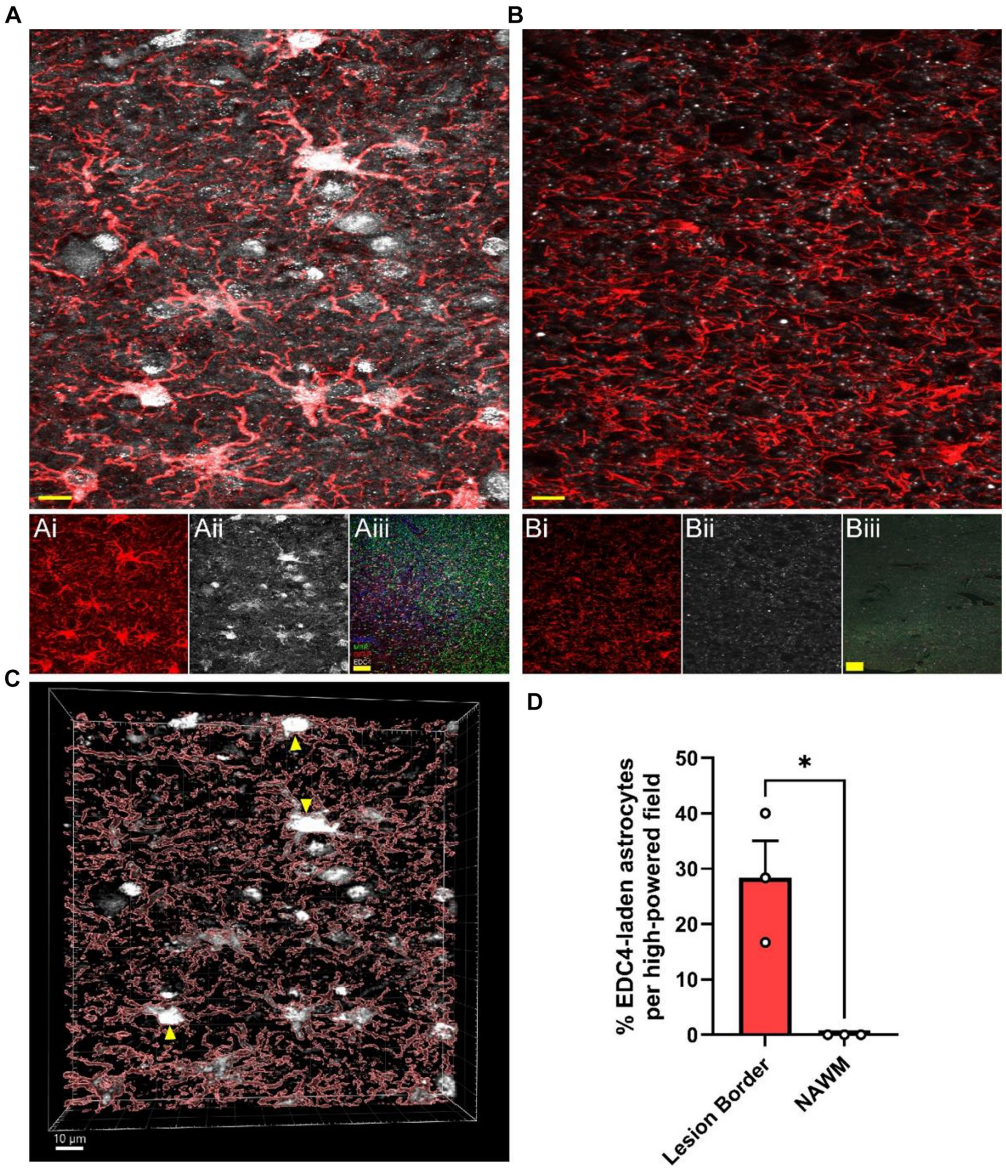
MS WMLs exhibit substantial EDC4 condensation. Representative merged confocal images of either **(A)** white matter lesion or **(B)** normal-appearing white matter stained for GFAP (red) and EDC4 (white). **(Ai,ii)** Single channel images of the representative chronic active lesion from MS patient 88. **(Aiii)** A representative merged 10X confocal image of the chronic active lesion characterization labeled for MHCII (Blue), MBP (Green), GFAP (Red), and EDC4 (White). **(Bi,ii)** Single channel images of the representative chronic active lesion from MS patient 88. **(Biii)** A representative merged 10X confocal image of tissue from patient 88 labeled for MHCII (Blue), MBP (Green), GFAP (Red), and EDC4 (White). Scale bars (yellow) in **(A)** and **(B)** are 10 μm. Scale bars (yellow) in **(Aiii)** and **(Biii)** are 100 μm. **(C)** 3D volumetric reconstruction of **(A)** showing buildup of EDC4 (white) in astrocytes (red; GFAP). Yellow arrowheads indicate EDC4-laden astrocytes. Scale bar is 10 μm. **(D)** Quantification of percent of astrocyte cell bodies laden with EDC4 per high powered field, data are representative of three patients, matched within patient, and 1–2 lesions were analyzed per patient. Statistics by two-sample *t* test. *p < 0.05.

Since MS lesions preferentially contain higher levels of *i2R-PSMB8* transcript compared to NAWM (Figure 1) and this transcript leads to enhanced P-body formation and NMD (Figures 2, 3), we next asked whether MS lesions also displayed an increase in P-body formation in astrocytes. To address this question, we obtained postmortem MS tissue sections and labeled for MBP and MHCII to determine lesion type along with GFAP and EDC4 to determine P-body burden within astrocytes. We chose to analyze chronic active WMLs due to their active lesion border that is dense with reactive astrocytes (Zeinstra et al., 2003). In confirmed chronic active lesion borders of MS patients (Figures 4Ai–iii), we observed striking densities of EDC4^+^ structures (Figure 4A), which appeared much larger than the P-bodies observed in our *in vitro* studies (Figures 2A,B). By contrast, much smaller EDC4^+^ structures were observed in MS patient NAWM (Figure 4B), which was distal to lesions and where MBP was apparently intact (Figures 4Bi–iii). Using 3D image rendering, we found that the large EDC4^+^ structures were frequently, but not exclusively, found within GFAP^+^ astrocytes (Figure 4C). Quantifying the number of these EDC4-laden astrocytes and normalizing to the number of GFAP^+^ cell bodies per high powered field, we observed an increasing trend in the proportion of EDC4-laden astrocytes in the chronic active lesion borders compared to in NAWM (Figure 4D; *p* = 0.0136). We also confirmed that this EDC4-laden phenotype occurs in MHC II^+^ cells (Supplementary Figure S5) and MBP^+^ cells (Supplementary Figure S6). These data indicate that the astrocytes, among other cells, of the chronic active lesion border undergo significant alternative splicing events, which are likely to the detriment of proper cellular function.

## Discussion

Multiple studies have shown a potential role for alternative splicing in neurodegenerative disorders (Paraboschi et al., 2014; De Rossi et al., 2016; Putscher et al., 2022; Shaw et al., 2022). In this study, we identified a novel *PSMB8* splice variant preferentially upregulated in demyelinated WMLs (Figure 1A), the primary pathology during MS. Further, our work demonstrates that this novel splice variant undergoes NMD and leads to an increase in P-body formation *in vitro* (Figures 2, 3). This preferential increase in a novel splice variant which is degraded in the WML led us to hypothesize that splicing may be globally dysregulated within the WMLs during MS. Indeed, we also found that large EDC4^+^ structures exist within cells of the WML, especially in astrocytes (Figure 4). These structures appeared to occupy a substantial volume of the GFAP^+^ cell bodies. These results suggest that in the lesion microenvironment, splicing is dysregulated within astrocytes which may further compound cellular stress.

Specifically, regarding the *i2R-PSMB8* isoform, this alternative splicing event may reduce the capacity for full induction of the immunoproteasome. While we did not observe changes in the total *PSMB8* expression in the WML environment, alterations in total expression are not a requisite for increased stress due to alternative splicing. Additionally, this novel *i2R-PSMB8* isoform is nonfunctional given its premature stop codon toward the beginning of the functional nucleophilic aminohydrolase domain (Figure 1C). The protein encoded by *PSMB8*, LMP7, is required for immunoproteasome assembly (Griffin et al., 1998). Loss of function of the immunoproteasome is detrimental in animal models of MS, such as EAE (Smith et al., 2020). Thus, this nonfunctional *i2R-PSMB8* isoform may represent a partial loss of induction of functional *PSMB8*. We note that while the *i2R-PSMB8* isoform appears to be a small portion of total in Figure 1A, this is actually an underestimation of the total amount made as we have now shown that it is substantially degraded through NMD. Whether the expression level of this isoform is enough to reduce the functional LMP7 below optimal levels remains unknown; however, the LMP7 protein concentration does closely correlate (*ρ* = 0.91) with the *PSMB8* mRNA counts, indicating a disruption in mRNA would likely have effects on functional protein (Jiang et al., 2020).

Beyond this specific isoform, though, it appears splicing as a whole may be dysregulated in the lesion microenvironment given the drastic increase in EDC4 labeling in MS lesions, as a single isoform is not likely to have such a profound impact. Proinflammatory cytokines induce alternative splicing in many genes *in vitro*, including in primary astrocytes derived from human MS patients (Werkman et al., 2020). Additionally, in animal models of MS, splicing is dysregulated in lesions and this aberrant splicing correlates with interleukin-1β expression (Marchese et al., 2021; Adinolfi et al., 2023). Further, the microglial splicing factor QKI-5 is downregulated in brain WMLs of MS patients (Lee et al., 2020). We show here that EDC4, a marker for NMD and a proxy for aberrant splicing, is highly upregulated in MS WMLs, frequently occurring in GFAP^+^ astrocytes, though other cells also show high levels of EDC4 expression. This most frequently occurred in the chronic active lesion border, where inflammation is thought to persist. This corresponds with preclinical observations correlating alternative splicing with inflammation (Marchese et al., 2021, Adinolfi et al., 2023). Indeed, a recent study has highlighted a potential role for the splicing factor hnRNP A1 in neurodegeneration in MS lesions (Salapa et al., 2024). In this study, Salapa et al. demonstrate that hnRNP A1 is mislocalized and changes its RNA binding in neurons in human MS brain lesions and spinal cord lesions of EAE mice. This alteration in hnRNP A1 localization and function led to increased alternative splicing in neuronal RNA and contributed to neuronal death (Salapa et al., 2024).

It is possible that in MS lesions, the immunoproteasome is one of possibly many responses to inflammation that could be dampened due to alternative splicing. We speculate that this increased alternative splicing in the lesion microenvironment may contribute to lesion progression or limit the ability of resident glia to repair damage. For instance, reactive astrocytes have impaired redox homeostasis (Sheng et al., 2013), and the EDC4-laden astrocytes in human MS lesions (Figure 4) are reactive and dystrophic. Furthermore, inhibition of the immunoproteasome leads to an increase in oxidatively damaged proteins in astrocytes *in vitro* and lesion expansion *in vivo* during EAE (Smith et al., 2020). Thus, splicing dysregulation may lead to EDC4-induced stress, resulting in astrocyte reactivity and loss of redox homeostasis, contributing to tissue damage. One limitation of our study is that we cannot determine whether the specific *i2R-PSMB8* transcript alone is sufficient to cause the substantial increase in EDC4 condensation observed in the MS lesion. We hypothesize that this one transcript alone is insufficient; however, the more likely explanation is that many genes are undergoing detrimental alternative splicing in the WMLs to give rise to this EDC4-laden phenotype. Further, the observed increases in P-body formation may result in increased cell stress and death, possibly contributing to the slowly expanding smoldering lesions frequently found in progressive MS. Identifying specific splicing factors affected in MS lesions could lead to a viable therapeutic strategy.

## Data availability statement

The original contributions presented in the study are included in the article/Supplementary material. Further inquiries can be directed to the corresponding authors, or further information can be found at this link: https://figshare.com/projects/A_novel_PSMB8_isoform_associated_with_multiple_sclerosis_lesions_induces_P-body_formation/203301.

## Ethics statement

Ethical approval was not required for the studies involving humans because cells were purchased from ScienCell Inc. and de-identified patient tissue used for this study did not qualify for IRB approval. The studies were conducted in accordance with the local legislation and institutional requirements. The human samples used in this study were acquired from gifted from another research group. Written informed consent to participate in this study was not required from the participants or the participants’ legal guardians/next of kin in accordance with the national and local legislation and the institutional requirements.

## Author contributions

BS: Conceptualization, Data curation, Formal analysis, Funding acquisition, Investigation, Methodology, Project administration, Software, Validation, Visualization, Writing – original draft, Writing – review & editing. JW: Funding acquisition, Methodology, Project administration, Resources, Supervision, Visualization, Writing – original draft, Writing – review & editing.
