# Supplementary material for: A novel PSMB8 isoform associated with multiple sclerosis lesions induces P-body formation
Source: Front Cell Neurosci. 2024 May 15;18:1379261. doi: 10.3389/fncel.2024.1379261 (PMC11133558; doi:10.3389/fncel.2024.1379261)
Supplement: Supplementary file 1 [file Data_Sheet_1.docx]

| **Gene** | **Forward Primer** | **Reverse Primer** |
| --- | --- | --- |
| *GBP2* | 5'-ACCCTCGCTCTTAAACTTCAG-3' | 5'-GGCTCCAATGATTTGCTTCTC-3' |
| *GGTA1* | 5'-CTCAGAAGGGCTGGTGGTTT-3' | 5'-TAAACCAGTCCCATAGCCGAAG-3' |
| *B3GNT5* | 5'-TGGTCGTGTTCATCGTGGTG-3' | 5'-CCGGCTGTGTAGTCAGGGTA-3' |
| *CD14* | 5'-CCGCTGTGTAGGAAAGAAGCTA-3' | 5'-ACAAGGTTCTGGCGTGGTC-3' |
| *PTX3* | 5'-TGCAGTGTTGGCCGAGAA-3' | 5'-GATGAAGAGCTTGTCCCATTCC-3' |
| *S100A10* | 5'-TCGCTGGGGATAAAGGCTACT-3' | 5'-AGCCCACTTTGCCATCTCTAC-3' |
| *TGM1* | 5'-GTCACCAACTTCAACTCCGC-3' | 5'-ATCCAGCAGTCGTTCCACAC-3' |
| *CD44* | 5'-ACGGAAGAAACAGCTACCCAG-3' | 5'-GGCTGGTATGAGCTGAGGC-3' |
| *GFAP* | 5'-CCTCTCCCTGGCTCGAATG-3' | 5'-GGAAGCGAACCTTCTCGATGTA-3' |
| *S1PR3* | 5'-CCCAGCCCATCTGGCATT-3' | 5'-CAGCGAGGGCGTTGAAAAAG-3' |
| *TIMP1* | 5'-CCAGACCACCTTATACCAGCG-3' | 5'-GGACCTGTGGAAGTATCCGC-3' |
| *CCL2* | 5'-AGGTGACTGGGGCATTGAT-3' | 5'-GCCTCCAGCATGAAAGTCTC-3' |
| *CCL5* | 5'-GAGTATTTCTACACCAGTGGCAAG-3' | 5'-TCCCGAACCCATTTCTTCTCT-3' |
| *CXCL12* | 5'-CAAGTGTGCATTGACCCGAA-3' | 5'-TACAAAGCGCCGAGAGCAA-3' |
| *GAPDH* | 5'-GAAGGTGAAGGTCGGAGTC-3' | 5'-GAAGATGGTGATGGGATTTC-3' |

**Supplemental Table 1.** Genes and associated primers used to analyze the data shown in Supplemental Figure 4.


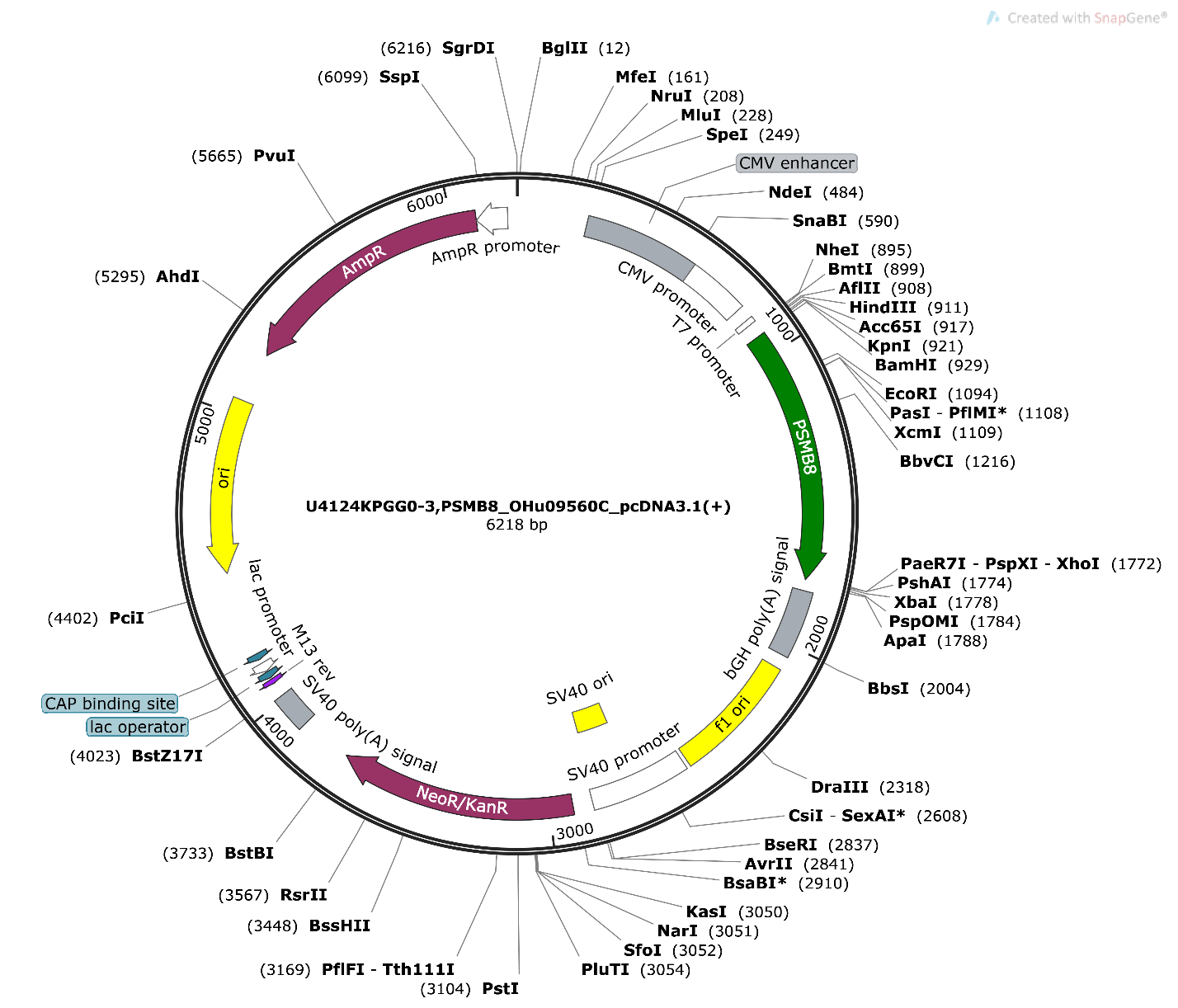


**Supplemental Figure 1.** Vector map of *FL-PSMB8* plasmid used in transfections. This plasmid was purchased from Genscript, catalog number SC1200, GenEZ ORF Clone: PSMB8_OHu09560C_pcDNA3.1(+). This is the canonical exon 1B containing *PSMB8*, NM_148919.4.


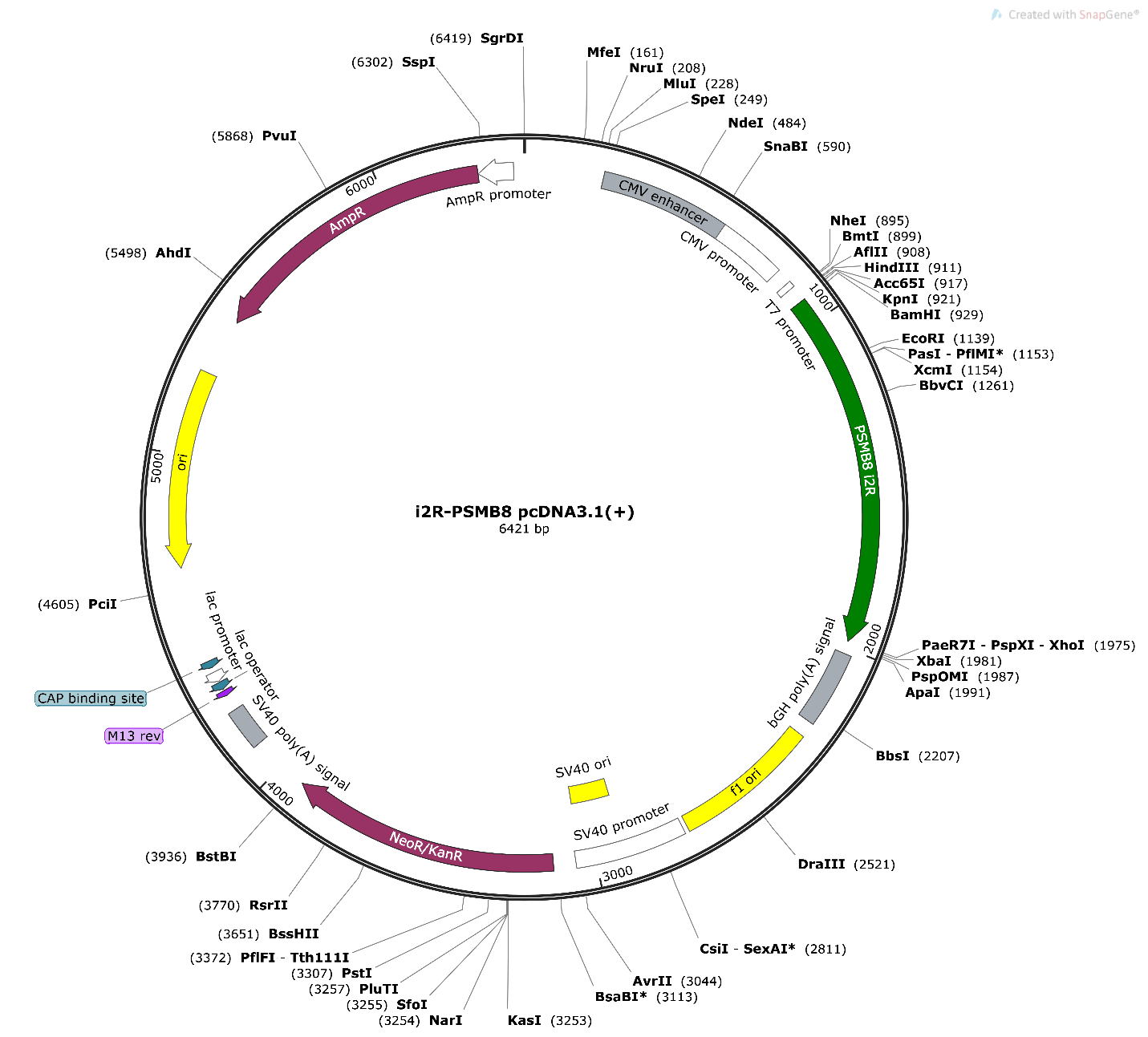


**Supplemental Figure 2.** Vector map of *i2R-PSMB8*. This was a custom gene synthesis from Genscript cloned into pcDNA3.1. This is identical cDNA as the exon 1B containing *PSMB8* but with intron 2 retained.


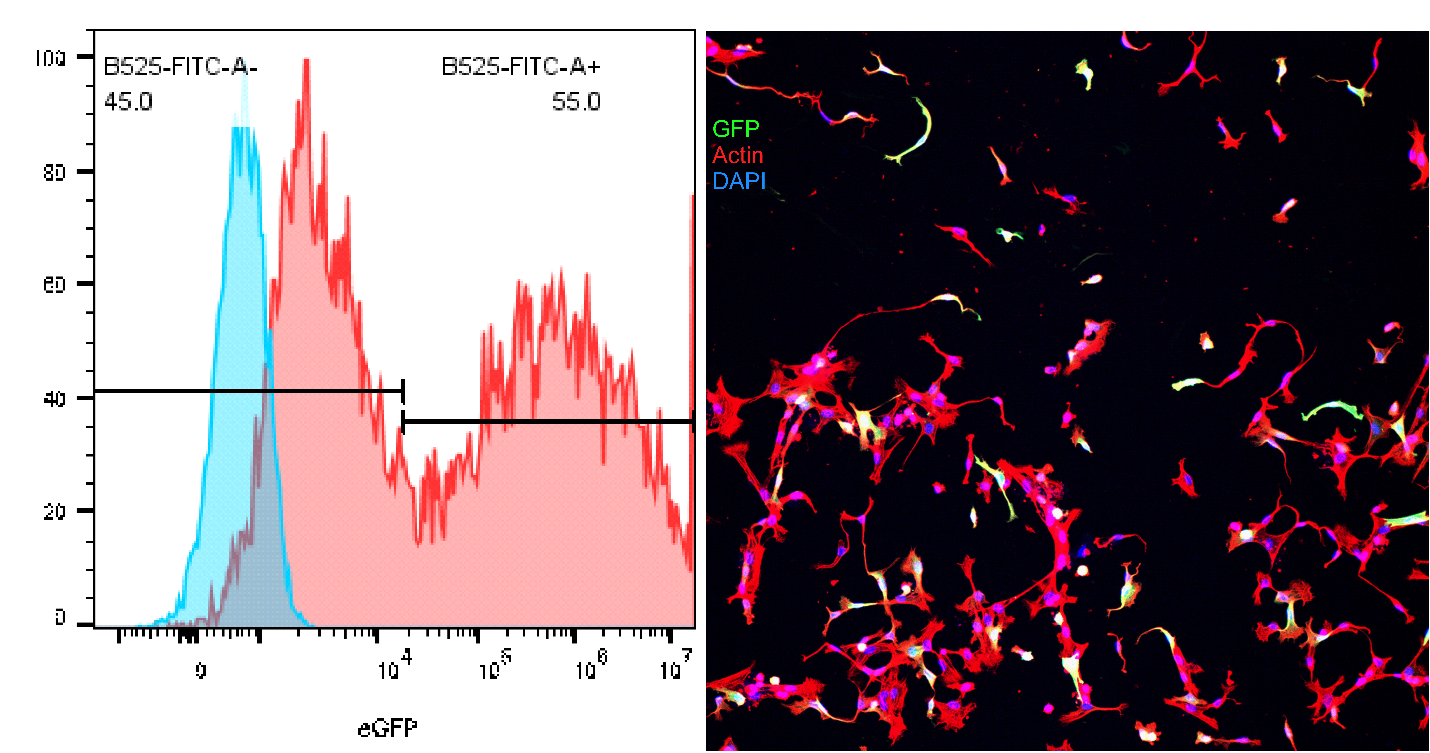


**Supplemental Figure 3.** Transfection efficiency of primary human cortical astrocytes. Left: flow cytometry of GFP-transfected primary human cortical astrocytes (red) or non-transfected controls (blue). Right: fluorescence microscopy image of GFP-transfected primary human cortical astrocytes. Actin is shown in red and DAPI in blue to mark all cells, and GFP, shown in green, as an indicator of transfection. Astrocytes sourced from ScienCell and transfections performed using a Lonza Nucleofector 4 device with 6 μg DNA per 10^6^ cells under program DR114.


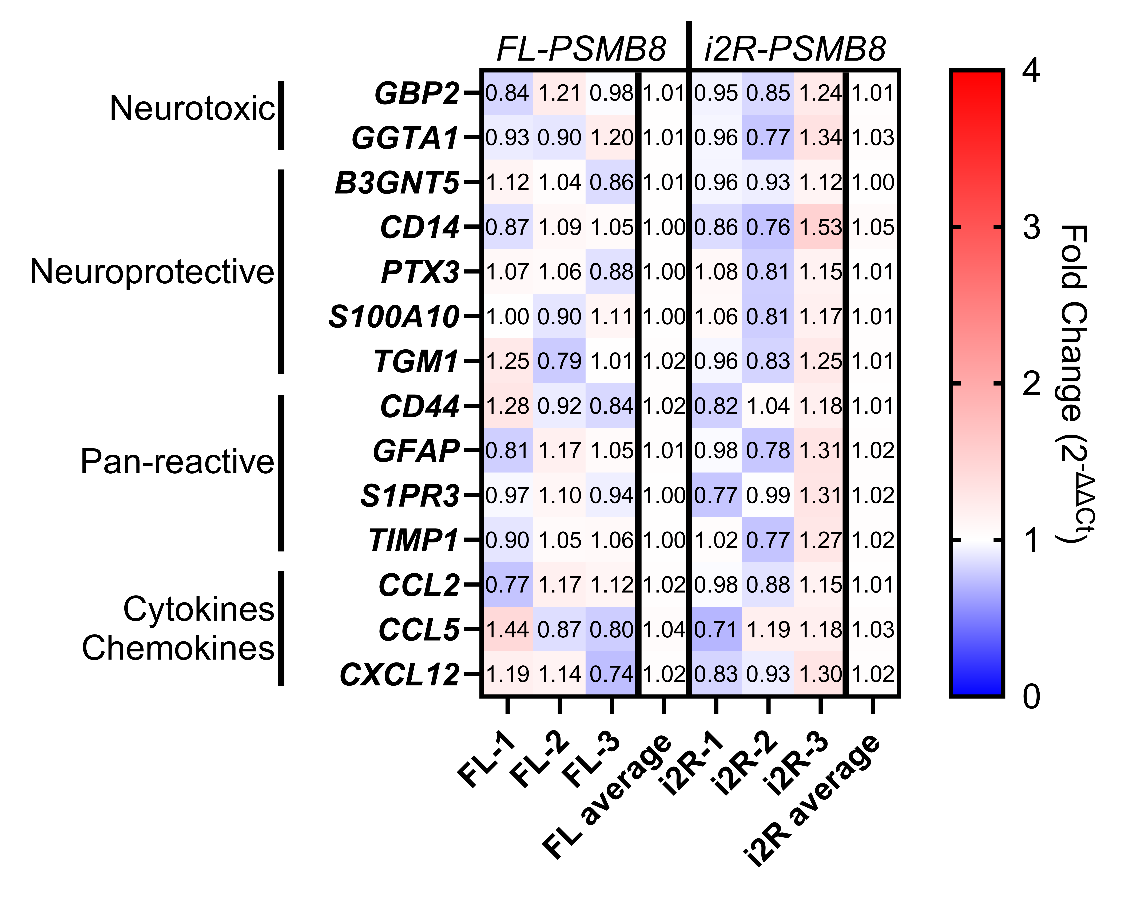


**Supplemental Figure 4.** Markers of astrocytic reactivity are unchanged at 24 hours post-transfection with the *i2R-PSMB8* vector compared to the *FL-PSMB8* vector. Individual transfection replicates are shown along with an average per group. Numbers in each cell indicate the corresponding fold change (2^-ΔΔCt^) for that sample or row average. No statistically significant changes were observed (FDR > 0.70 for all genes).


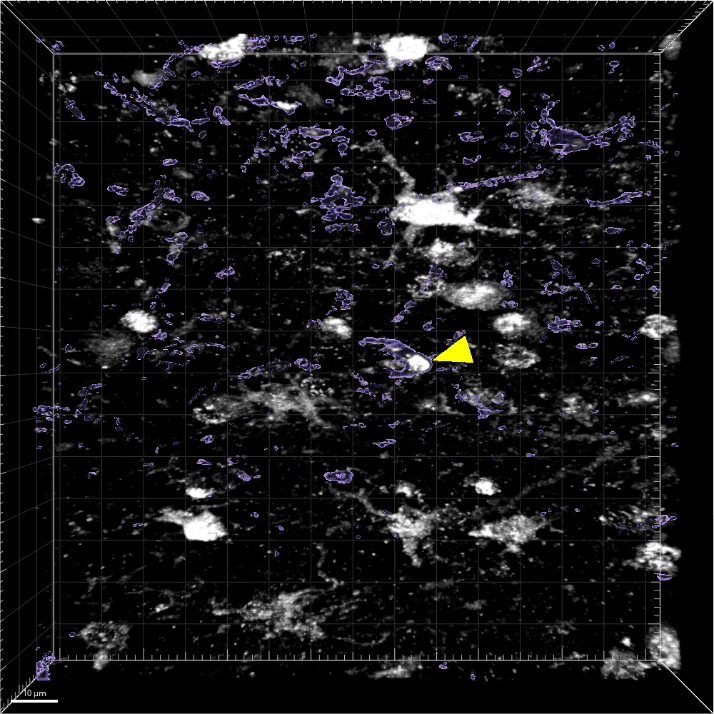

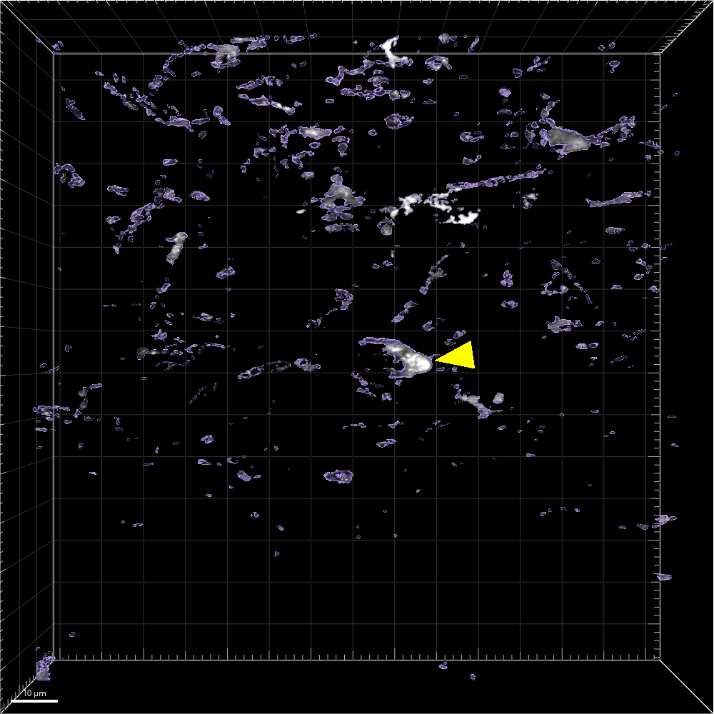


**Supplemental Figure 5.** EDC4 condensation in MHC-II^+^ cell. Left: All EDC4 shown. Right: Only EDC4 colocalized with MHC-II shown. Blue: MHC-II. White: EDC4. Image from white matter lesion border. Yellow arrow indicates EDC4-laden cell. Scale bar is 10 μm.


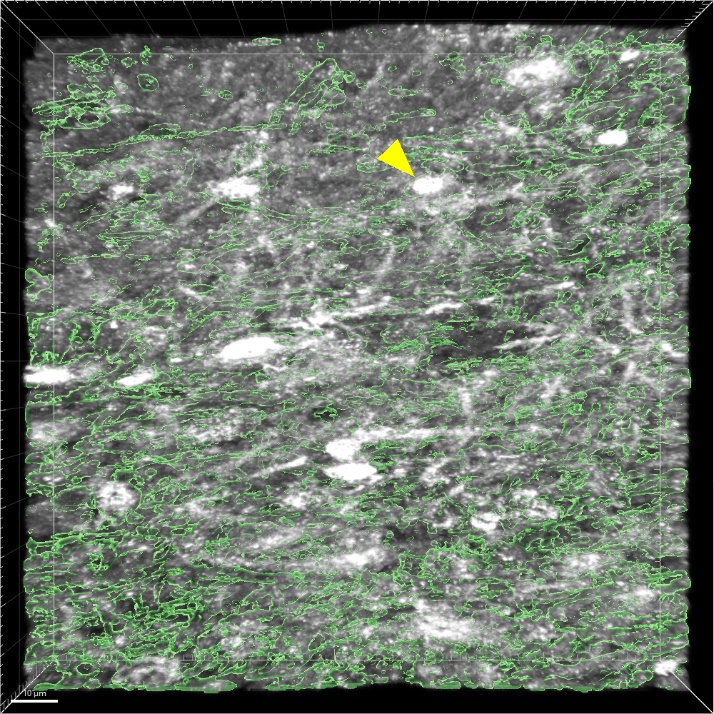

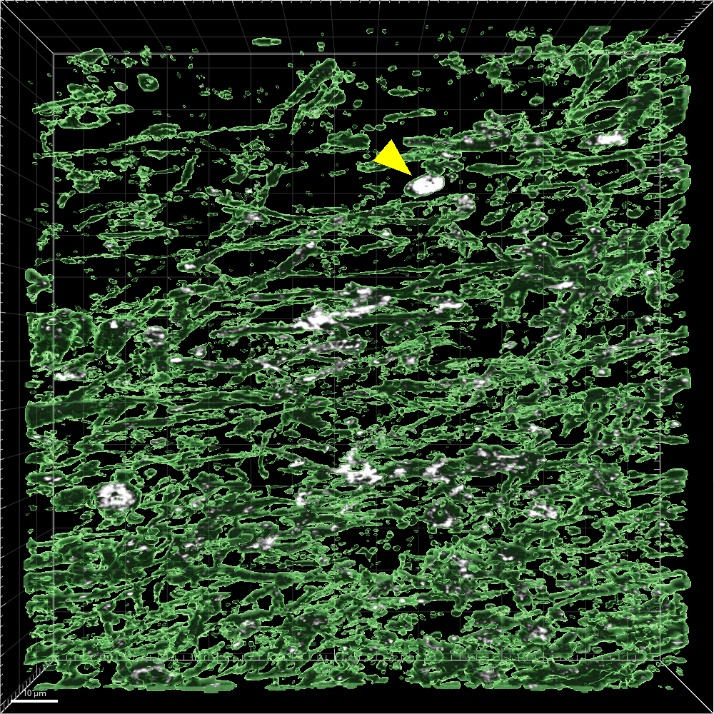


**Supplemental Figure 6.** EDC4 condensation in MBP^+^ cell. Left: All EDC4 shown. Right: Only EDC4 colocalized with MBP shown. Green: MBP. White: EDC4. Image from white matter lesion border. Yellow arrow indicates EDC4-laden cell. Scale bar is 10 μm.
